# Green Extraction Strategies for Orange Peel Dust Valorization with Enhanced Bioactive Potential

**DOI:** 10.3390/foods15091495

**Published:** 2026-04-25

**Authors:** Isidora Vlaović, Slađana Krivošija, Vanja Travičić, Ivana Mitrović, Gordana Ćetković, Aleksandra Gavarić, Senka Vidović

**Affiliations:** Faculty of Technology Novi Sad, University of Novi Sad, Boulevard Cara Lazara 1, 21000 Novi Sad, Serbia; isidora.vlaovic@uns.ac.rs (I.V.); sladjana.krivosija@uns.ac.rs (S.K.); vanjaseregelj@uns.ac.rs (V.T.); tadi@uns.ac.rs (I.M.); gcetkovic@uns.ac.rs (G.Ć.); cvejina@uns.ac.rs (A.G.)

**Keywords:** citrus by-products, subcritical water extraction, pressurized ethanol extraction, ultrasound-assisted extraction, supercritical CO_2_, circular bioeconomy, enzyme inhibition, bioactive recovery

## Abstract

Despite its rich bioactive composition, orange peel dust (OPD), a fine industrial by-product generated during citrus processing in the filter tea industry, has not received much attention as a valuable matrix. Using antioxidant activity (2,2-diphenyl-1-picrylhydrazyl (DPPH), 2,2′-azino-bis-(3-ethylbenzothiazoline-6-sulfonic acid) (ABTS), and reducing power (RP)), α-amylase inhibitory activity, antimicrobial potential, and sugar composition as function-oriented indicators, this study aimed to compare four green extraction technologies: subcritical water extraction (SWE), pressurized ethanol extraction (PEE), ultrasound-assisted extraction (UAE), and sequential supercritical CO_2_–UAE (Sc-CO_2_–UAE) applied to OPD derived from *Citrus sinensis* L. Among thermally driven techniques, PEE at 220 °C had the highest radical-scavenging activity, while UAE showed the broadest antifungal activity against *Fusarium* spp. and *Alternaria alternata*, along with selective antibacterial activity against *Bacillus cereus*. Sequential Sc-CO_2_ pretreatment at 300 bar followed by UAE resulted in the highest α-amylase inhibitory activity. Sugar analysis indicated that thermal conditions enhanced carbohydrate hydrolysis, while UAE and Sc-CO_2_-UAE maintained structural sugars under mild conditions. All green extraction approaches outperformed conventional maceration. These findings validate OPD as a valuable industrial by-product suitable for sustainable valorization, supporting circular economy concepts in the citrus processing sector.

## 1. Introduction

In recent years, the principles of circular economy and sustainable development have gained prominence in global policy frameworks and industrial practice. While global efforts increasingly focus on achieving the United Nations Sustainable Development Goals (SDGs) by 2030, challenges related to environmental pollution, inefficient resource use, and food waste generation remain unresolved. The food manufacturing sector is among the major contributors to global waste streams, with fruits representing the largest fraction of waste generated during the manufacturing stage [1,2].

Among fruit commodities, oranges (*Citrus sinensis* L.) represent the most processed citrus fruit worldwide, with annual global production exceeding 76 million metric tons [3]. Orange processing waste generates large quantities of by-products, primarily peels, which account for approximately 60–65% of the total fruit mass, alongside seeds and internal tissues such as pulp and membranes [4]. Zema et al. [5] reported that the worldwide annual output of orange peel waste is approximately ten million metric tons. As a result, orange peel has attracted significant scientific attention as a valuable source of bioactive compounds and functional ingredients [5,6,7].

However, in addition to the conventionally studied coarse peel fractions, industrial processing of dried orange peel, particularly within the filter tea industry, generated a substantial amount of fine residue known as orange peel dust (OPD). This fraction may account for up to 35% of the processed material and consists of particles smaller than 0.315 mm, which are below the pore size of standard filter paper and therefore unsuitable for further processing using conventional production steps [8]. Due to its unfavorable handling properties and limited compatibility with standard downstream operations, OPD is commonly classified as industrial waste rather than a usable raw material [8,9].

Unlike coarse orange peel, OPD represents a technologically distinct matrix characterized by extremely fine particle size, high specific surface area, and altered mass behavior. These properties directly influence extraction kinetics, solvent accessibility, and diffusion phenomena, thereby justifying OPD as a separate and relevant model system for extraction process evaluation. Despite being treated as waste, OPD has received increased scientific interest as a rich source of biologically active compounds such as flavonoids, polyphenols, pectin, dietary fiber, essential oils, and organic acids [9]. Many of these compounds have been associated with antioxidant, antimicrobial, anti-inflammatory, neuroprotective, and metabolic health–related effects, highlighting their potential relevance for food, pharmaceutical, and cosmetic applications [10,11,12].

Furthermore, the fine structure and large surface-to-volume ratio of OPD make it a suitable model matrix for the comparative evaluation of modern green extraction technologies. Such characteristics can enhance mass transfer, reduce solvent and energy requirements, and accentuate differences between extraction approaches, including subcritical water extraction (SWE), pressurized ethanol extraction (PEE), ultrasound-assisted extraction (UAE), and supercritical carbon dioxide extraction (Sc-CO_2_) [8,13]. Therefore, the valorization of OPD through green extraction techniques is not only environmentally justified but also scientifically relevant for process optimization and potential industrial upscaling.

In response to environmental concerns and circular economy principles, green extraction technologies have been increasingly explored for the recovery of bioactive compounds from citrus waste [14,15]. Compared to conventional extraction methods, which often suffer from low efficiency, prolonged processing times, thermal degradation of thermolabile compounds, and reliance on hazardous organic solvents, modern green approaches offer improved selectivity, reduced environmental impact, and enhanced sustainability [7,16]. These technologies are closely aligned with the SDGs, particularly those related to food security, human health, clean water, sustainable energy use, and responsible consumption and production (SDGs 2, 3, 6, 7, and 12) [17].

Systematic comparative evaluations of multiple green extraction technologies applied to the same OPD matrix, assessed through complementary biological assays, remain scarce. Moreover, the potential of OPD as a model substrate for integrated and sequential green extraction strategies has not been sufficiently explored. To further illustrate this gap, Table 1 provides a comparative overview of selected studies on green extraction of bioactive compounds from citrus peel matrices. As shown, available experimental studies predominantly investigate a single extraction technique applied to conventional peel fractions, with biological activities limited to antioxidant capacity or phenolic profiling [18,19]. Studies that incorporate a broader range of biological activities, such as antimicrobial activity and digestive enzyme modulation, still rely on a single extraction approach and do not address OPD as a distinct industrial subfraction [20]. Based on the available literature, no published study has simultaneously compared SWE, PEE, UAE, and sequential Sc-CO_2_–UAE on OPD, while integrating antioxidant, α-amylase inhibitory, and antimicrobial activities alongside sugar composition as complementary indicators of extract functionality.

In this context, the present study focuses on the systematic evaluation of OPD as a distinct industrial residue within the framework of green extraction and circular economy principles. It is hypothesized that the unique physicochemical structure and chemical composition of OPD enable selective and efficient recovery of functionally relevant compounds depending on the applied extraction technology. Therefore, this work aims to comparatively assess subcritical water extraction, pressurized ethanol extraction, ultrasound-assisted extraction, and sequential supercritical CO_2_–UAE processes, using antioxidant, antimicrobial, and α-amylase inhibitory activities together with sugar composition as indicators of extract functionality and potential industrial applicability.

## 2. Materials and Methods

### 2.1. Plant Material

OPD derived from *Citrus sinensis* L. was obtained from a local filter tea production facility (Fructus d.o.o., Bačka Palanka, Serbia), where it is generated as a by-product during cutting, grinding, and fractionation of dried orange peel. The collected OPD fraction consisted of particles smaller than 0.315 mm. The moisture content of the material was determined gravimetrically and amounted to 3.36 ± 0.63%.

### 2.2. Chemicals and Reagents

2,2-diphenyl-1-picryhydrazyl (DPPH), 2,2′-azino-bis-(3-ethylbenzothiazoline-6-sulfonic acid) diammonium salt (ABTS), methanol (>99%), ferric chloride, trichloroacetic acid, and Trolox were purchased from Sigma Chemical Company (St. Louis, MO, USA), and α-amylase was purchased from Sigma-Aldrich (Buchs, Switzerland). Ethanol (96%) was purchased from Zorka Pharma (Šabac, Serbia). Potassium ferricyanide, sodium dihydrogen phosphate dihydrate, disodium hydrogen phosphate dodecahydrate, and all other chemicals used in the assays were of analytical grade or higher.

### 2.3. Extraction Techniques

**Figure 1:**
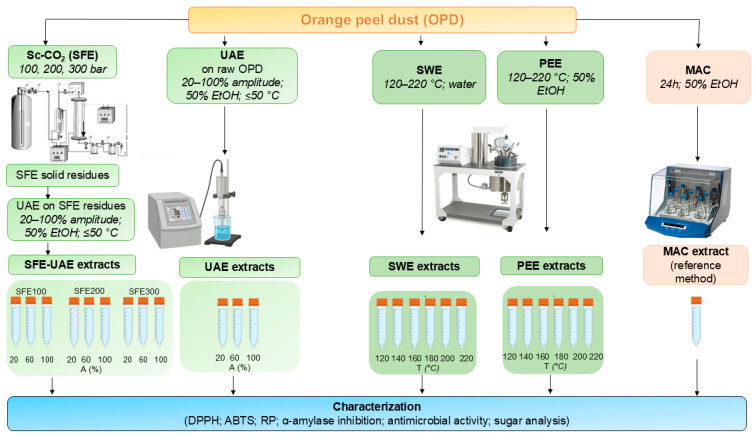
Schematic representation of the extraction procedures applied to obtain orange peel dust (OPD) extracts. Abbreviations: SWE: subcritical water extraction; PEE: pressurized ethanol extraction; UAE: ultrasound-assisted extraction; SFE–UAE: supercritical CO_2_ extraction followed by ultrasound-assisted extraction; MAC: maceration.

OPD extracts evaluated in the present study were obtained using green extraction technologies described by Krivošija et al. [22,23]. The overall experimental procedures are schematically summarized in Figure 1. The resulting extracts were subsequently subjected to biological and chemical characterization.

Two different green extraction strategies were applied. In the first approach, supercritical CO_2_ extraction (Sc-CO_2_) was performed at pressures of 100, 200, and 300 bar, for 4 h, resulting in three supercritical OPD extracts and corresponding solid residues (SFE-100, SFE-200, and SFE-300). These residues obtained after Sc-CO_2_ extraction were subsequently subjected to ultrasound-assisted extraction (UAE) using a 50% (*v*/*v*) ethanol/water as solvent with varying ultrasound amplitude (20, 60, and 100%). A 50% (*v*/*v*) ethanol–water mixture was selected as the extraction solvent due to its balanced polarity, which enables the extraction of both hydrophilic and moderately polar compounds from the OPD matrix. Ethanol–water systems in this range have been widely reported as suitable for the recovery of phenolic compounds from citrus by-products, with optimal extraction often observed at around 50% ethanol [24]. This is in agreement with previous studies on orange peel, where 50% ethanol showed as an effective solvent for ultrasound-assisted extraction of antioxidants [25]. In addition, UAE of untreated raw OPD (not previously subjected to Sc-CO_2_ extraction) was carried out using the same solvent system to evaluate the efficiency of process integration. UAE was conducted until the extraction temperature reached 50 °C to preserve bioactive compounds. Conventional maceration (MAC) for 24h using the same solvent system was additionally performed as a reference method to enable comparison with the green extraction techniques.

In the second approach, subcritical water extraction (SWE) and pressurized ethanol extraction (PEE) were conducted. Same solid-to-solvent ratio of 1:20 (*w*/*v*) was applied for SWE, PEE, UAE, and MAC, in accordance with previously reported studies [22,23]. For SWE, 7 g of OPD were extracted with 140 mL of double-distilled water, while for PEE a 50% (*v*/*v*) ethanol solution was used as the extraction solvent. Temperature (120–220 °C) was applied as the independent variable for both extraction types, while pressure (20 bar) and extraction time (15 min) were kept constant.

All extractions were performed in triplicate (independent experiments), ensuring reproducibility of the extraction process.

### 2.4. Antioxidant Activity Assays

The antioxidant potential of the extracts was investigated spectrophotometrically using three assays: DPPH, ABTS and reducing power (RP) on different wavelengths (515 nm, 414 nm, and 700 nm respectively). All measurements were performed in triplicate. Results were expressed as mmol Trolox equivalents (TE) per 100 g of dried sample.

Spectrophotometric measurements were performed using a MultiskanGO microplate reader (Thermo Fisher Scientific Inc., Waltham, MA, USA).

#### 2.4.1. DPPH Assay

The protocol for the DPPH assay was performed according to the method described by Aborus et al. [26]. A DPPH stock solution was prepared at a concentration of 0.35 mg/mL in methanol and diluted to an absorbance of approximately 1.0 at 515 nm before use. A 96-well microplate was filled with 250 µL of the prepared DPPH solution and 10 µL of the diluted samples. The plate was then kept in the dark for 50 min, after which the absorbance was measured at 515 nm, using methanol as the blank.

#### 2.4.2. ABTS Assay

ABTS scavenger activity was evaluated employing a modified method according to Šeregelj et al. [27]. An ABTS stock solution was prepared at a concentration of 5.5 mg/mL in bidistilled water and activated by the addition of MnO_2_, followed by filtration. The activated solution was diluted to an absorbance of approximately 1.0 at 414 nm before use. A volume of 250 μL of the prepared ABTS solution was added to 2 μL of the sample, with absorbance recorded at 414 nm after 35 min. Water was used as a blank, and absorbances were calculated using Equation (1).

Δ*A* = *A*_0_ − *A_fin_* − *A_blank_* (1)

where:

A_0_ is the initial absorbance of the activated ABTS solution
A_fin_ is the final absorbance after incubation for 35 min
A_blank_ is the absorbance of the blank (water).

#### 2.4.3. Reducing Power (RP)

RP was determined using a 96-well microplate adaptation of the method described by Oyaizu [28]. Briefly, 75 μL of potassium iron (III) cyanide (1%), 75 μL sodium phosphate buffer (pH = 6.6), and 75 μL of appropriately diluted sample solution or 75 μL of extractant (blank) were mixed. The solutions were heated in a water bath for 20 min at 50 °C and then cooled to room temperature. Subsequently, 75 μL of 10% trichloroacetic acid was added, followed by centrifugation. After centrifugation, 50 µL of distilled water and 10 µL of 0.1% ferric chloride solution were added to 50 µL of the collected supernatant. Absorbances were measured immediately at a wavelength of 700 nm. A calibration curve was constructed with Trolox, and the results were expressed as mmol Trolox equivalent per ml of sample. The resulting calibration curve equation has the form:

RP = (A700/5229) × 1000/250.29(2)

Then the RP of the sample was expressed as mM Trolox equivalents in 100 g of dried sample (mM TEAC/100 g of sample).

### 2.5. Determination of α-Amylase Inhibitory Activity

The inhibitory activity against α-amylase was evaluated using a colorimetric method based on the quantification of reducing sugars released from starch, following the principle of the dinitrosalicylic acid (DNS) assay. The determination of α-amylase inhibitory activity was performed according to the method of Šavikin et al. [29], with minor modifications.

Briefly, 200 μL of diluted extract was mixed with 200 μL of α-amylase solution (0.10 mg/mL). Both extracts and enzyme solutions were prepared in phosphate buffer (0.1 M, pH 6.9). After incubation at 37 °C for 15 min, 200 μL of 1% (*m*/*v*) starch solution prepared in the same buffer was added, and the mixture was further incubated for 10 min at 37 °C. The reaction was stopped by adding 200 μL of DNS reagent, followed by heating in a boiling water bath for 15 min. After cooling, absorbance was measured at 540 nm. Acarbose was used as a positive control, and the percentage inhibition of α-amylase activity by the extracts was compared to the inhibition achieved by acarbose.

### 2.6. Sugar Analysis

Extracts evaluated in the present study were clarified by filtration through a 0.2 μm Chromafil filter (Macherey-Nagel, Duren, Germany) and subsequently subjected to HPLC analysis to determine the amount of present sugars [30]. Chromatographic measurements were performed using a Thermo Scientific Dionex UltiMate 3000 UHPLC (Thermo Fisher Scientific, Waltham, MA, USA) system equipped with HPG-3200SD/RS pump, a WPS-3000(T) SL autosampler with a 10 µL injection loop, HPLC catrige guard column (Agilent Technologies, Santa Clara, CA, USA), and a RefractoMax 520 (ERC, Tokyo, Japan) refractive index detector.

In accordance with data from scientifically available literature [27,31,32], it was established that orange peel contains several non-structural sugars. Before analysis, a calibration curve was constructed using selected sugars based on the relevant scientific literature. The calibration standards included: glucose, fructose, sucrose, arabinose, galactose, xylose, rhamnose, mannose, maltose, and raffinose. Acetonitrile (75%, *v*/*v*) was used as eluent at a flow rate of 1.2 mL/min and an elution time of 15 min. Zorbax NH2 (250 mm × 4.6 mm, 5 μm) column (Agilent Technologies, Santa Clara, CA, USA) was used as the stationary phase, at a constant temperature of 25 ± 0.5 °C. The samples were analyzed in three replicates (*n* = 3).

### 2.7. Antimicrobial Activity

OPD extracts obtained under different experimental conditions were tested for their antifungal and antibacterial activity. Fungal strains known for their significant mycotoxin production were selected as test organisms, including *Fusarium graminerum*, *Fusarium avenaceum*, *Alternaria alternata,* and *Aspergillus flavus*. The diffusion method with wells was used for in vitro testing in Petri dishes, and 100 μL of the corresponding extract was used for testing [33]. After 10 days of incubation at 27 °C, the diameters of mycelial growth inhibition zones were measured. All diameters greater than 22 mm are an indicator that the applied extract is highly effective on the tested fungus [34].

On the other hand, one representative of Gram-positive bacteria, *Bacillus cereus*, and one representative of Gram-negative bacteria, *Salmonella enterica* subsp. *enterica* ATCC 13076, were selected for testing antibacterial activity. The disc diffusion method was employed using sterile paper discs (HiMedia, Mumbai, India), onto which 10 μL of the appropriate extract was applied [35]. After incubation for 72 h at a temperature of 30 °C, the diameters of the inhibition zones were measured using HiAntibiotic ZoneScale (HiMedia, Mumbai, India) ruler. According to this method, inhibition zone diameters larger than 11 mm were considered indicative of high antibacterial activity [30]. Sterile distilled water and 50% (*v*/*v*) ethanol were used as a negative control. All experiments were performed in triplicate.

### 2.8. Statistical Analysis

All experimental results are presented as mean ± standard deviation (SD) of three independent measurements. Statistical differences among samples were evaluated using one-way analysis of variance (ANOVA), followed by Tukey’s post hoc test. Differences were considered statistically significant at *p* < 0.05.

## 3. Results and Discussion

### 3.1. Antioxidant Activities

The antioxidant capacity of complex extracts cannot be fully characterized by a single method due to the diverse chemical nature and mechanisms of action of individual antioxidant compounds. Therefore, in this study, three complementary in vitro assays, namely DPPH, ABTS, and RP, were applied to evaluate the antioxidant properties of the obtained extracts. To improve clarity of antioxidant responses under different extraction conditions, the results for DPPH, ABTS, and RP assays are presented in Table 2.

The chemical profile of OPD extracts, including the identification of hesperidin, naringin, narirutin, and rutin as dominant flavonoids, along with data on extraction yields under comparable experimental conditions, has been established in our previous studies [22,23]. Therefore, this work focuses on the comparative evaluation of the biological activities of the applied extraction techniques.

#### 3.1.1. DPPH Radical Scavenging Activity

Although the applied extraction techniques differ substantially in their operating principles, several general trends can be observed. The antioxidant activity of both SWE and PEE extracts increased consistently with rising extraction temperature. A similar upward trend was observed for UAE extract with increasing ultrasound amplitude and pretreatment pressure. The DPPH activities reported in the literature for *Citrus sinensis* L. peel extracts [36] are of the same order of magnitude as those obtained in the present study, indicating comparable antioxidant potential.

Among all tested extracts, PEE obtained at 220 °C exhibited the highest DPPH radical-scavenging activity and was statistically superior to all other treatments. This behavior is consistent with the enhanced solubilization capacity of ethanol–water mixtures at elevated temperatures, which enables the extraction of a broader range of antioxidant compounds, including those of intermediate polarity [37]. Since SWE and PEE were conducted under identical conditions, differing only in the extraction solvent, the observed differences in antioxidant activity can be attributed to solvent-related selectivity properties. At lower extraction temperatures, SWE extracts exhibited higher DPPH activity than PEE, likely due to the high polarity of subcritical water and its strong affinity for polar phenolic antioxidants [38,39].

Regarding UAE extracts, increasing ultrasound amplitude resulted in higher antioxidant capacity, which can be associated with more intense cavitation and mechanical disruption of the plant matrix [40]. In addition, higher pretreatment pressure during Sc-CO_2_ extraction was followed by increased DPPH activity in subsequent UAE extracts, suggesting that process intensity plays an important role in antioxidant recovery. Although UAE applied directly to untreated OPD generally resulted in slightly higher DPPH activity, the application of UAE to residues remaining after Sc-CO_2_ represents a notable advantage from a sustainability perspective, as it enables further valorization of extraction residues and supports a zero-waste approach.

Overall, all extracts obtained using green technologies outperformed conventional maceration, confirming the superior efficiency of green approaches for the recovery of DPPH-active antioxidants from OPD.

#### 3.1.2. ABTS Radical Scavenging Activity

Consistent with the trends observed in the DPPH assay, antioxidant capacity significantly increased with rising extraction temperatures and process intensity across all applied methods. Notably, ABTS values were substantially higher than those obtained in the DPPH assay. This observation may be related to the higher sensitivity of the ABTS assay towards hydrophilic antioxidant compounds, which are detected more efficiently compared to the DPPH method [41,42].

Within the PEE series, ABTS radical-scavenging activity exhibited a pronounced temperature-dependent increase reaching the highest level at 220 °C. A similar, though slightly less pronounced, trend was observed for SWE, with antioxidant activity increasing steadily with temperature. The higher ABTS activity observed for PEE compared to SWE at elevated temperatures is likely related to the presence of ethanol, which enhances the solubilization of phenolic compounds of intermediate polarity compared to water alone.

In the UAE, extracts obtained from raw OPD, a marked increase in ABTS activity when ultrasound amplitude was increased to 100%, indicating the importance of cavitation intensity for antioxidant recovery. Moreover, UAE applied to residues remaining after extraction proved to be particularly effective. This finding suggests that high-pressure Sc-CO_2_ may modify the OPD matrix structure and enhance the accessibility of antioxidant compounds during subsequent extraction steps. Such pretreatment is likely to facilitate solvent penetration and diffusion of polar antioxidants that were previously associated with lipophilic domains of the matrix [43,44].

Overall, all extracts obtained using green extraction technologies exhibited significantly higher ABTS radical-scavenging activity than conventional maceration, highlighting the superior efficiency of green approaches for the recovery of ABTS-active antioxidants and supporting their applicability for the sustainable valorization of OPD biomass.

#### 3.1.3. RP Assay

Among all tested samples, the highest reducing power was observed for PEE extracts obtained at elevated temperatures, with maximum activity recorded at 220 °C, indicating the strong influence of thermal conditions on the recovery of compounds associated with reducing power. A similar temperature-dependent increase was observed for SWE extracts up to 200 °C, followed by a slight decrease at 220 °C. This trend suggests that while subcritical water at higher temperatures may promote the extraction or formation of redox-active compounds, excessively severe conditions may affect specific phenolic structures responsible for electron-donating capacity. In contrast, PEE extracts exhibited a continuous increase in reducing power with rising temperature, likely reflecting the enhanced ability of ethanol–water mixtures to solubilize phenolic compounds with strong reducing potential. Compared to thermally driven extraction techniques, UAE applied directly to raw OPD resulted in relatively modest changes in reducing power across different ultrasound amplitudes. This observation indicates that increasing ultrasound intensity mainly facilitates the overall release of antioxidants, as reflected by ABTS activity, but does not substantially increase the fraction of constituents exhibiting high electron-donating capacity. However, when UAE was applied to residues remaining after Sc-CO_2_ extraction, a gradual increase in reducing power was observed with increasing pretreatment pressure. The highest reducing power among UAE-derived extracts was obtained after pretreatment at 300 bar, suggesting that the combined SFE–UAE approach enhances the accessibility of redox-active compounds and promotes a more comprehensive utilization of the OPD matrix.

The reducing power observed for OPD extracts is consistent with literature reports describing citrus peels as rich sources of phenolic compounds with pronounced ferric-reducing activity [6]. Although direct numerical comparison is limited by differences in plant species and experimental conditions, the magnitude of reducing power values obtained in this study is comparable to those reported for other citrus matrices, such as *Citrus unshiu* peels [19]. The reducing capacity detected in UAE-derived extracts may be partly associated with the presence of phenolic compounds, including flavonoids, which have previously been identified as major constituents of orange peel dust [22], and are known to contribute to antioxidant activity in citrus by-products, as reported in previous studies [45].

### 3.2. Antidiabetic Activity

The inhibitory activity against α-amylase of OPD extracts obtained using different extraction strategies is presented in Table 3. Overall, pronounced differences in enzyme inhibition were observed among the tested samples, indicating a strong influence of extraction conditions on the recovery and preservation of enzyme-interacting constituents.

The highest inhibition was recorded for extracts obtained using sequential SFE–UAE processing, with the extract derived from material pretreated at 300 bar and subsequently subjected to UAE at 100% amplitude showing the strongest inhibitory effect. Extracts obtained after pretreatment at 200 and 100 bar, followed by UAE, also exhibited high inhibition levels. Among single-step extraction approaches, SWE performed at moderate temperature showed notable inhibitory activity, whereas further increases in SWE temperature resulted in a pronounced decline in enzyme inhibition.

UAE applied directly to raw OPD also showed considerable inhibitory effects, indicating that the peel matrix naturally contains compounds capable of interacting with carbohydrate-hydrolysing enzymes. However, the enhanced inhibitory activity observed for sequential SFE–UAE extracts highlights the importance of process integration, suggesting that the combined approach may improve α-amylase inhibitory activity.

The observed differences in α-amylase inhibition among OPD extracts are consistent with the established role of citrus by-products as a source of phenolic compounds with potential relevance for the modulation of carbohydrate-hydrolyzing enzymes [45,46]. Citrus peels are particularly rich in flavonoids, including hesperidin, naringin, and related compounds, whose recovery strongly depends on solvent polarity and extraction conditions. In this context, the variation observed between SWE, PEE, UAE, and sequential SFE-UAE extracts likely reflects differences in extraction selectivity rather than only differences in total extracted material. Similar observations have been reported for citrus peel extracts, where extraction conditions influence both phytochemical composition and inhibitory activity against digestive enzymes [46].

From a mechanistic perspective, the present results should be interpreted with caution. The observed α-amylase inhibitory activity may be associated with the presence of phenolic compounds, including flavonoids, as suggested by literature data [47], and supported by previously reported TPC and TFC values for UAE and SFE-UAE extracts [22]. It is well established that flavonoids can inhibit α-amylase activity, with their effectiveness depending on structural features such as hydroxylation pattern, glycosylation, and molecular conformation [47,48]. Accordingly, the higher inhibition observed in certain OPD extracts may reflect differences in the recovery of compounds that interact with enzymes under specific extraction conditions [46,47,48]. However, since individual bioactive compounds were not characterized in the present study, the results support only an association between extraction conditions and α-amylase inhibitory potential, rather than definitive attribution of activity to specific compounds. This interpretation is consistent with previous studies in which antidiabetic activity is discussed in relation to the overall phytochemical profile rather than to a single identified constituent [46,47,48].

Overall, the obtained results indicate that OPD can serve as a promising source of extracts with potential relevance for the development of functional ingredients with α-amylase inhibitory properties.

### 3.3. Sugar Profile

Orange peel contains a diverse profile of carbohydrates. These sugars originate from both soluble intracellular components and cell wall, making orange peel a valuable carbohydrate-rich agro-industrial by-product [49]. The analysis of free sugars was performed to provide additional insight into the chemical changes occurring within the OPD matrix under different extraction conditions. As major primary metabolites in plant tissues, sugars are particularly sensitive to thermal processing and can undergo hydrolysis, degradation, or transformation at elevated temperatures, making them useful indicators of process-induced modifications [50,51]. An increase in extraction temperature during SWE and PEE in the range of 120–220 °C significantly affects the sugar composition of orange peel extracts. At moderate temperatures (120–160 °C), partial hydrolysis of polysaccharides occurs, leading to an increased release of soluble sugars, primarily glucose and fructose derived from saccharose, and arabinose, galactose, and xylose derived from cellulose, hemicellulose, and pectin fractions [52,53]. At higher temperatures (≥180 °C), thermal degradation of released sugars becomes more pronounced. Monosaccharides undergo dehydration and fragmentation reactions, resulting in the formation of furanic compounds such as 5-hydroxymethylfurfural and furfural, accompanied by a decrease in detectable sugar concentrations [54]. This is especially pronounced in the PEE type of extraction (Table 4).

As shown in Table 4, glucose, fructose, xylose, arabinose, and sucrose were detected in extracts obtained at lower and moderate temperatures. In the SWE series, the concentrations of glucose and fructose increased up to 160 °C, which is consistent with sucrose hydrolysis and partial depolymerization of structural carbohydrates under subcritical water conditions [55]. At higher temperatures, a gradual decrease in total sugar content was observed. A similar trend was observed for PEE extracts, where free sugars were detected up to 200 °C, while no sugars were detected at 220 °C, suggesting more extensive thermal transformation under the most severe extraction conditions.

The reduction in free sugar content at elevated extraction temperatures coincided with a pronounced increase in ABTS radical-scavenging activity. These findings indicate that high-temperature extraction processes promote chemical transformations within the OPD matrix that influence the overall functional profile of the obtained extracts.

Orange peel carbohydrates are predominantly present in the form of structural polysaccharides, including pectin, cellulose, and hemicellulose, which generally require elevated temperatures, acidic conditions, or prolonged extraction times to undergo depolymerization into soluble monosaccharides. The sugar profile obtained after UAE revealed a selective recovery of structural monosaccharides rather than common soluble sugars such as glucose, fructose, or sucrose. Ultrasound-assisted extraction primarily relies on acoustic cavitation, which enhances mass transfer through mechanical disruption of plant tissues while operating under relatively mild thermal conditions [13].

At 20% amplitude, arabinose, galactose, and mannose were detected. suggesting partial solubilization of hemicellulosic and pectic side chains. Fishman et al. [56] reported that galactose and arabinose are major neutral sugars present in citrus pectin side chains. Increasing the ultrasound amplitude to 60% led to the appearance of xylose. The emergence of xylose at higher amplitudes can be explained by a greater disruption of hemicellulosic components of the plant cell wall. At the highest amplitude (100%), xylose increased, indicating that high-energy ultrasound treatment intensifies the disruption of hemicellulosic structures and promotes the release of xylan-derived fragments. At the same time, the relatively stable concentration of galactose across all amplitudes suggests a continuous release of galactan-rich fragments from pectic polysaccharides during progressive cell wall disintegration.

Therefore, the absence of glucose, fructose, and sucrose suggests that UAE did not promote significant hydrolytic cleavage of structural carbohydrates or sucrose breakdown. Instead, the detected sugars likely originated from pre-existing soluble fractions or from partial mechanical disintegration of hemicellulosic and pectic side chains. This selective behavior distinguishes UAE from high-temperature extraction methods and supports its suitability for the recovery of bioactive compounds under non-destructive processing conditions [13].

In contrast to SWE and PEE, the SFE–UAE extracts (samples 16–24) were characterized predominantly by sucrose as the major detected sugar, while fructose and glucose were present in lower concentrations and pentoses were detected only in minor amounts or traces (Table 4). This profile reflects the non-hydrolytic nature of Sc-CO_2_ pretreatment and the moderate polarity of the 50% ethanol system applied during UAE, which primarily favors the extraction of soluble sugars rather than structural polysaccharide-derived monosaccharides.

Increasing ultrasound amplitude did not result in enhanced release of structural sugars; instead, a reduction in total free sugar content was observed at 100% amplitude, suggesting possible degradation or transformation under intensified cavitation conditions [13].

The higher sugar content observed in sequential SFE–UAE extracts compared to UAE alone can be explained by the structural modifications induced during the supercritical CO_2_ pretreatment. Although supercritical CO_2_ is non-polar and does not directly extract carbohydrates, it efficiently removes lipophilic components such as essential oils, waxes, and other hydrophobic constituents from the plant matrix. This removal reduces structural barriers within the cell wall and increases matrix porosity. As a result, the subsequent ultrasound-assisted extraction is performed on a more permeable and structurally loosened material, facilitating improved solvent penetration and enhanced mass transfer. The intensified accessibility of hemicellulosic and pectic domains likely promotes the release of structurally bound monosaccharides during UAE [57].

These findings highlight the importance of pretreatment in cascade extraction systems and demonstrate how non-polar supercritical extraction can indirectly improve the recovery of polar compounds in subsequent processing steps.

Since maceration represents a mild extraction technique that does not significantly disrupt the structural polysaccharides of the plant cell wall, the detected sugars (glucose, fructose and sucrose) most likely originate from freely soluble carbohydrates rather than from the depolymerization of pectins or hemicelluloses. Under such conditions, the appearance of structural monosaccharides such as arabinose, galactose, xylose, or rhamnose is generally limited, as these sugars are mainly bound within complex cell wall polysaccharides and require stronger mechanical, thermal, or chemical treatments for their release [58].

### 3.4. Antimicrobial Activity of OPD Extracts

Orange peel is a rich source of bioactive phenolic compounds, including flavonoids (hesperidin and naringin) and phenolic acids such as *p*-coumaric, gallic, and caffeic acids [58], which have been extensively reported for their antimicrobial potential against phytopathogenic fungi and foodborne bacteria [59].

For antimicrobial activity evaluation, all extracts listed in Table 2 were tested. However, only extracts exhibiting antimicrobial activity are presented in Table 5. Therefore, the influence of extraction method and processing intensity was evaluated based on this subset of samples.

Regarding the antimicrobial activity of the tested extracts, they exhibit both antifungal and antibacterial effects. The observed differences in antifungal activity among extracts obtained by SWE, UAE, and PEE can be primarily attributed to variations in extraction selectivity, solvent polarity, and the chemical profile of bioactive compounds recovered from *C. sinensis* peel. Antifungal activity was observed against *Fusarium* isolates and *A. alternata*, whereas no activity was detected against *A. flavus*. This outcome is expected, as *Fusarium* spp. and *A. alternata* possess cell membranes that are particularly susceptible to phenolic compounds and terpenoids, which can disrupt membrane integrity, inhibit ergosterol biosynthesis, and interfere with mitochondrial function. In contrast, *A. flavus* is known to exhibit higher intrinsic resistance, largely due to its robust antioxidant defense system, efficient efflux pumps, and its ability to metabolize or detoxify phenolic compounds [60]. Farouk et al. [61] similarly reported that *C. sinensis* L. peel essential oil exhibits pronounced antifungal activity against *Fusarium* species, while no activity was observed against *A. flavus*. On the other hand, Samandari-Najafabadi et al. [62] confirmed the antifungal effects of *C. sinensis* essential oil against *Alternaria* pathogens.

Results show that UAE demonstrated the broadest and most consistent antifungal activity, particularly against *F. graminearum*, *F. avenaceum,* and *A. alternata*, especially when higher amplitudes (60% and 100%) were employed. This can be explained by the cavitation effect induced by ultrasound, which enhances cell wall disruption and facilitates the release of intracellular and cell wall–bound secondary metabolites, including flavonoids, phenolic acids, and terpenoids [13]. These compounds are well known for their antifungal properties. Moreover, UAE operates under mild temperature conditions, minimizing thermal degradation of thermolabile antifungal compounds and preserving their biological activity.

With regard to antibacterial activity, the results presented in Table 5 indicate that the samples obtained by UAE exhibited the desired antibacterial effect against the Gram-positive bacterium *B. cereus*. In contrast, none of the analyzed samples showed antagonistic activity against the Gram-negative bacterium *S. enterica*. The selective activity against *B. cereus* can be attributed to structural differences between Gram-positive and Gram-negative bacteria, particularly the absence of an outer membrane in Gram-positive bacteria, which facilitates the penetration of bioactive compounds present in the extracts. Conversely, the outer lipopolysaccharide membrane of Gram-negative bacteria acts as an effective barrier, limiting the susceptibility of *S. enterica* to these compounds. These findings are consistent with the results reported by Ngan et al. [63], who investigated the antibacterial activity of orange essential oils obtained by hydrodistillation and solvent-free microwave extraction. Their study shows that among all tested gram-positive and gram-negative bacteria, antibacterial activity was observed exclusively against *B. cereus*.

Overall, the results of this study demonstrate that orange peel represents a valuable source of antimicrobial compounds, whose biological activity is strongly influenced by the extraction technique applied. Among the evaluated methods, ultrasound-assisted extraction proved to be the most effective approach, yielding extracts with broad and consistent antifungal activity against *Fusarium* spp. and *A. alternata*, as well as selective antibacterial activity against *B. cereus*.

### 3.5. Comparative Assessment of Extraction Strategies and Functional Potential of OPD Extracts

To further interpret the performance of the applied extraction strategies, the obtained results were evaluated in the context of green extraction approaches applied to agro-industrial by-products. Fruit- and plant-derived residues commonly used in food processing industries represent relevant model matrices, as their structural characteristics and chemical composition strongly influence extraction efficiency and biological activity. In such systems, techniques such as subcritical water extraction, pressurized liquid extraction, ultrasound-assisted extraction, and supercritical CO_2_ processing have been shown to differently affect the recovery of bioactive compounds and the resulting functional properties. A comparative overview of representative studies, together with the results obtained in the present study, is presented in Table 6, enabling qualitative assessment of extraction performance across different matrices and processing conditions.

The selected extraction techniques represent distinct green extraction approaches based on different driving mechanisms (thermal, mechanical, and supercritical), enabling a direct comparison of their performance on the same OPD matrix and across different agro-industrial residues. As shown in Table 6, consistent trends can be observed across different matrices and extraction techniques, highlighting the strong influence of extraction conditions on the functional profile of the obtained extracts. For SWE, both the present study and the literature indicate that higher extraction temperatures favor antioxidant recovery, whereas enzyme-inhibitory activity is maximized at lower-to-moderate temperatures. In the study by Liu et al. [64], the highest antioxidant activity (DPPH IC_50_ = 0.83 mg/mL) was obtained at 220 °C, while the strongest α-amylase and α-glucosidase inhibition was observed at 120 °C. A comparable trend was obtained for OPD, where antioxidant activity increased with temperature (8.75–13.16 mM TEAC/100 g), whereas α-amylase inhibition peaked at 62.96% at 160 °C and decreased at higher temperatures. This shift in the temperature optimum for enzyme inhibition likely reflects differences in extract composition and matrix-specific extraction behavior.

A similar temperature-dependent behavior was observed for PEE. In both the present study and the literature, pressurized hydroethanolic extraction resulted in high antioxidant capacity [65], with literature data reporting values of 371.00 μmol TE/g dw alongside high phenolic content. In the OPD system, antioxidant activity increased with temperature, reaching a maximum at 220 °C, whereas α-amylase inhibition peaked at 37.36% at 160 °C and declined at higher temperatures. This divergence suggests that different groups of compounds contribute to antioxidant activity and enzyme inhibition, with their extraction being differently affected by temperature and extraction conditions. It should be noted that Poblete et al. [65] did not assess enzyme-inhibitory activity, and antimicrobial activity was not evaluated, limiting direct comparison of functional potential across the two systems.

For UAE, both the present study and the literature indicate its suitability for the recovery of bioactive compounds from plant-based matrices. As reported in [66], UAE enabled efficient extraction of phenolic compounds, reaching high antioxidant activity (DPPH 151.16 mM Trolox/g dw; ABTS 360.67 mM Trolox/g dw), with optimal performance observed at moderate ultrasound intensity. Antimicrobial activity was also reported against *Escherichia coli* and *Staphylococcus aureus*. In the OPD system, antioxidant activity showed only a slight increase with increasing ultrasound amplitude, whereas α-amylase inhibition remained relatively high across all conditions. In addition, UAE extracts exhibited antifungal activity against *Fusarium* spp. and *Alternaria alternata*, as well as selective antibacterial activity against *Bacillus cereus*. These findings suggest that, in contrast to thermally driven techniques, ultrasound intensity plays a less pronounced role in modulating antioxidant response, while still enabling effective recovery of compounds associated with antimicrobial and enzyme-inhibitory activity, although direct comparison is constrained by inherent differences in chemical composition between the investigated matrices.

A distinct behavior was observed for the sequential SFE–UAE approach. In the study by Domínguez-Rodríguez et al. [67], different extraction steps yielded fractions with distinct functional profiles, with direct UAE using NaDES showing the highest antioxidant activity (TEAC and ORAC assays), while SFE fractions were enriched in terpenoid compounds and exhibited higher anticholinesterase activity (AChE and BChE inhibition), highlighting the complementary nature of the sequential process. In the OPD system, the sequential approach resulted in the highest α-amylase inhibitory activity among all tested techniques (79.47%), whereas antioxidant activity remained moderate (DPPH up to 8.00 mM TEAC/100 g; ABTS up to 68.44 mM TEAC/100 g). These findings indicate that, unlike thermally driven extraction techniques, the SFE–UAE process does not maximize overall antioxidant capacity, but rather favors the recovery of compounds associated with enzyme inhibition. This behavior can be attributed to the selective removal of non-polar components during SFE, followed by enhanced accessibility of more polar bioactive compounds during the subsequent UAE step. These observations further support the relevance of OPD as a distinct agro-industrial matrix, whose fine particle size and increased surface area contribute to enhanced extraction efficiency and differential response to applied green extraction techniques.

The choice of extraction technique directly determines process feasibility, energy demand, and scalability of OPD valorisation [58]. Thermally driven solvent systems (SWE and PEE) enable efficient recovery of polar and semi-polar compounds due to enhanced solvation and mass transfer at elevated temperatures [58,68]. In contrast, UAE promotes process intensification through cavitation, reducing extraction time and energy demand [13]. Supercritical CO_2_ extraction, although capital-intensive, provides solvent-free extracts and high selectivity towards non-polar compounds [57,58]. The integration of supercritical CO_2_ pretreatment with subsequent extraction techniques, as demonstrated in this study, has been reported to improve overall extraction efficiency [57].

Techno-economic assessments performed on citrus processing residues have demonstrated that the choice of extraction technology significantly affects production costs and overall process feasibility, with production costs reported in the range of 5–7 USD/kg depending on process configuration and energy integration [69]. Although these values refer to citrus residues, they provide a relevant benchmark for OPD valorisation due to comparable processing characteristics. From a sustainability perspective, energy consumption represents an important parameter in evaluating extraction processes. Although a quantitative energy assessment was beyond the scope of this study, the applied techniques are widely recognized as green extraction approaches due to reduced solvent consumption, shorter processing times, and improved efficiency compared to conventional methods [37,70,71].

A key advantage of OPD is its inherently fine particle size (<0.315 mm), which facilitates mass transfer and minimizes the need for additional mechanical pretreatment, thereby reducing energy input and improving overall process efficiency. Based on the obtained results, thermally driven techniques (SWE and PEE) appear more suitable when antioxidant recovery is targeted, whereas UAE shows greater potential for applications requiring antimicrobial activity, while sequential SFE–UAE is more suitable when enhanced enzyme-inhibitory effects are targeted. This indicates that the techno-economic feasibility of OPD valorisation is strongly dependent on the intended application, with different extraction strategies offering advantages depending on the targeted functional outcome.

Future research should focus on process scale-up and optimization under industrially relevant conditions, supported by design of experiments (DoE) and process integration strategies to improve efficiency, reproducibility, and scalability. In parallel, the stability of OPD-derived extracts during processing and storage should be systematically evaluated. Further studies are required to validate the biological relevance of the observed in vitro activities through in vivo models and bioavailability assessments. The development of delivery systems, such as encapsulation or incorporation into functional food and nutraceutical formulations, represents a promising strategy to enhance stability and functionality.

## 4. Conclusions

This study demonstrates that OPD, a fine industrial by-product generated during citrus processing, represents a suitable matrix for the application and comparative evaluation of green extraction technologies, allowing the production of extracts exhibiting antioxidant, antimicrobial, and α-amylase inhibitory activities. The results confirm that extraction conditions and process integration strongly influence the recovery of bioactive compounds and the biological activity of the obtained extracts.

For thermally driven techniques, both SWE and PEE showed a clear increase in antioxidant activity as a function of temperature. The highest radical-scavenging activity and reducing power were obtained for PEE extracts at 220 °C, indicating that elevated temperature in combination with the ethanol-water solvent system improves the recovery of antioxidant compounds. At the same time, the observed decrease in detectable sugars at higher temperatures suggests that carbohydrate degradation and temperature-dependent changes in extract composition may contribute to the observed antioxidant properties.

UAE also showed a strong dependence on process intensity, with increasing ultrasound amplitude improving antioxidant recovery. Sequential extraction combining Sc-CO_2_ pretreatment and UAE further improved the accessibility of bioactive compounds. In particular, extracts obtained after Sc-CO_2_ pretreatment at 300 bar, followed by UAE, resulted in the highest α-amylase inhibitory activity, indicating that the applied extraction sequence may enhance the recovery of compounds associated with α-amylase inhibitory activity.

Sugar analysis showed that extraction conditions significantly affect carbohydrate composition. SWE and PEE at high temperatures promote hydrolysis and subsequent degradation of sugars, while UAE mainly facilitates the release of structural monosaccharides under mild conditions without extensive hydrolytic transformations. In SFE-UAE extracts, sucrose remained the dominant sugar, reflecting the non-hydrolytic character of Sc-CO_2_ pretreatment and the selective extraction of soluble carbohydrates during the UAE step.

In addition to antioxidant properties, OPD extracts exhibited antifungal activity against *Fusarium species* and *Alternaria alternata* and selective antibacterial activity against *Bacillus cereus*. Overall, the results highlight the potential of OPD as an underutilized industrial residue that can be effectively valorized through green extraction technologies. Such an approach supports circular economy principles by reducing residues from the filter tea industry and promoting their conversion into bioactive extracts and functional ingredients.

## Figures and Tables

**Table 1:** Comparison of the present study with representative literature on citrus peel valorization using green extraction techniques.

Study	Matrix	Extraction Methods	Biological Endpoints	Key Distinction vs. Present Study
Present study OPD	(*C. sinensis*, <0.315 mm, filter tea industry)	SWE, PEE, UAE, Sc-CO_2_–UAE	DPPH; ABTS; RP; α-amylase inhibition; antimicrobial; sugar composition;	Novel matrix; full multi-technology + multi-endpoint integration; sequential strategy;
Kim & Lim [21]	Standard peel, *C. unshiu*	SWE only (semi-continuous)	DPPH; FRAP; ORAC; XO; ACE; α-glucosidase; PL inhibition;	Single technique; different species and standard peel fraction (not OPD); no α-amylase inhibition, no antimicrobial activity, no sugar analysis;
Lachos-Perez et al. [18]	Standard orange peel, *C. sinensis*	Sequential SWE only	Flavanones; sugar composition;	Single technique; no UAE, PEE, or Sc-CO_2_–UAE; no antioxidant, antimicrobial, or α-amylase endpoint; coarse peel fraction, not OPD;
Durmus et al. [12]	Citrus peel (review)	Various	Phenolic compounds; antioxidant activity;	Review study; no systematic comparison on a single matrix; no α-amylase inhibition, antimicrobial, or sugar analysis;
Gómez-Urios et al. [20]	Standard orange peel, *C. sinensis*	NADES extraction (single technique)	Phenolic profile; antioxidant and antimicrobial; digestive enzyme inhibition;	Single extraction technique (NADES); coarse peel fraction, not OPD; no SWE, PEE, UAE, or Sc-CO_2_–UAE; no sugar composition analysis;

**Table 2:** Summary of antioxidant activity of OPD extracts obtained under different extraction conditions (mM TEAC/100g).

Sample Code	DPPH (mM TEAC/100 g)	ABTS (mM TEAC/100 g)	RP (mM TEAC/100 g)
SWE 120	8.75 ± 0.03 ^c, D–E^	16.22 ± 0.00 ^f, P^	4.07 ± 0.00 ^e, F–G^
SWE 140	9.27 ± 0.01 ^c, D^	24.98 ± 0.01 ^e, L–O^	5.20 ± 0.01 ^d, D^
SWE 160	10.88 ± 0.02 ^b, C^	41.16 ± 0.00 ^d, J^	7.52 ± 0.00 ^c, C^
SWE 180	12.48 ± 0.02 ^a, B^	48.50 ± 0.03 ^c, H–I^	10.04 ± 0.02 ^b, B^
SWE 200	12.93 ± 0.01 ^a, B^	61.09 ± 0.17 ^b, E–F^	11.71 ± 0.06 ^a, A^
SWE 220	13.16 ± 0.00 ^a, B^	71.25 ± 0.20 ^a, B–C^	10.52 ± 0.00 ^b, B^
PEE 120	7.76 ± 0.00 ^d, E–I^	21.82 ± 0.00 ^e, O–P^	3.08 ± 0.01 ^e, H^
PEE 140	7.83 ± 0.01 ^d, E–H^	24.17 ± 0.01 ^e, M–O^	3.30 ± 0.00 ^e, G–H^
PEE 160	8.68 ± 0.00 ^d, D–E^	43.90 ± 0.00 ^d, I–J^	5.12 ± 0.01 ^d, D^
PEE 180	10.47 ± 0.01 ^c, C^	52.95 ± 0.01 ^c, G–H^	7.48 ± 0.01 ^c, C^
PEE 200	12.19 ± 0.01 ^b, B^	75.30 ± 0.01 ^b, B–C^	10.38 ± 0.00 ^b, B^
PEE 220	14.74 ± 0.05 ^a, A^	90.91 ± 0.06 ^a, A^	11.81 ± 0.01 ^a, A^
UAE-20	7.97 ± 0.01 ^a–b, E–G^	28.44 ± 0.08 ^c–e, K–N^	4.61 ± 0.02 ^a–c, D–F^
UAE-60	8.01 ± 0.02 ^a–b, E–G^	31.45 ± 0.09 ^c–d, K–L^	4.65 ± 0.02 ^a–b, D–F^
UAE-100	8.17 ± 0.04 ^a, D–F^	53.51 ± 0.15 ^b, G–H^	4.99 ± 0.00 ^a, D–E^
SFE100-UAE20	6.57 ± 0.02 ^d, I^	22.22 ± 0.01 ^f, N–P^	4.08 ± 0.01 ^c, F–G^
SFE100-UAE60	6.74 ± 0.02 ^c–d, H–I^	26.50 ± 0.01 ^d–f, K–O^	4.51 ± 0.01 ^a–c, D–F^
SFE100-UAE100	7.37 ± 0.00 ^a–d, F–I^	29.95 ± 0.01 ^c–d, K–M^	4.61 ± 0.02 ^a–c, D–F^
SFE200-UAE20	6.91 ± 0.01 ^c–d, G–I^	23.41 ± 0.00 ^e–f, N–O^	4.23 ± 0.04 ^b–c, E–F^
SFE200-UAE60	7.18 ± 0.00 ^b–d, F–I^	27.17 ± 0.01 ^d–f, K–O^	4.65 ± 0.04 ^a–b, D–F^
SFE200-UAE100	7.20 ± 0.00 ^b–d, F–I^	32.84 ± 0.02 ^c, K^	4.98 ± 0.00 ^a, D–E^
SFE300-UAE20	7.57 ± 0.02 ^a–c, E–I^	56.02 ± 0.16 ^b, F–G^	4.86 ± 0.02 ^a, D–F^
SFE300-UAE60	7.82 ± 0.01 ^a–b, E–H^	64.66 ± 0.18 ^a, D–E^	4.88 ± 0.02 ^a, D–E^
SFE300-UAE100	8.00 ± 0.00 ^a–b, E–G^	68.44 ± 0.19 ^a, C–D^	5.03 ± 0.00 ^a, D^
MAC	6.60 ± 0.03 ^I^	9.37 ± 0.01 ^Q^	4.50 ± 0.00 ^D–F^

Different letters indicate statistically significant differences at *p* < 0.05. Lowercase letters represent differences within individual extraction series (e.g., SWE or PEE across temperatures, UAE across amplitudes), while uppercase letters denote differences among all tested extraction techniques. All results are expressed as mean values ± standard deviation of three independent determinations (*n* = 3). Numbers indicate process parameters: UAE—amplitude (%); SFE—pressure (bar); SWE/PEE—temperature (°C). SWE—subcritical water extraction; PEE—pressurized ethanol extraction; UAE—ultrasound-assisted extraction; SFE-UAE—solid residues—ultrasound-assisted extraction; MAC—maceration.

**Table 3:** α-Amylase inhibition (%) of OPD extracts.

Sample Code	Inhibition (%)
SWE 120	38.29 ^bc, H–J^
SWE 140	40.63 ^b, HI^
SWE 160	62.96 ^a, B–D^
SWE 180	33.65 ^c, JK^
SWE 200	43.36 ^b, H^
SWE 220	6.25 ^d, M^
PEE 120	31.54 ^b, K^
PEE 140	31.28 ^b, K^
PEE 160	37.36 ^a, H–K^
PEE 180	35.87 ^a, I–K^
PEE 200	19.42 ^c, L^
PEE 220	13.42 ^d, L^
UAE-20	53.60 ^ef, FG^
UAE-60	56.97 ^de, D–F^
UAE-100	59.05 ^c–e, D–F^
SFE100-UAE20	55.26 ^ef, FG^
SFE100-UAE60	62.71 ^cd, C–E^
SFE100-UAE100	65.61 ^bc, BC^
SFE200-UAE20	57.50 ^de, D–F^
SFE200-UAE60	50.35 ^f, G^
SFE200-UAE100	69.38 ^b, B^
SFE300-UAE20	56.45 ^d–f, E–G^
SFE300-UAE60	56.97 ^de, D–F^
SFE300-UAE100	79.47 ^a, A^
MAC	59.52 ^C–F^

Different letters indicate statistically significant differences at *p* < 0.05. Lowercase letters represent differences within individual extraction series (e.g., SWE or PEE across temperatures, UAE across amplitudes), while uppercase letters denote differences among all tested extraction techniques. All results are expressed as mean values ± standard deviation of three independent determinations (*n* = 3). Numbers indicate process parameters: UAE—amplitude (%); SFE—pressure (bar); SWE/PEE—temperature (°C). SWE—subcritical water extraction; PEE—pressurized ethanol extraction; UAE—ultrasound-assisted extraction; SFE-UAE—solid residues—ultrasound-assisted extraction; MAC—maceration.

**Table 4:** Identified sugars in OPD extracts (%).

Extract Type	Sugar	Concentration (%) ± SD
SWE 120	Xylose	0.2632 ± 0.0011
	Fructose	0.4545 ± 0.0003
	Glucose	0.6835 ± 0.0002
	Sucrose	0.4543 ± 0.0002
SWE 140	Xylose	0.2487 ± 0.0002
	Fructose	0.5461 ± 0.0003
	Glucose	0.7639 ± 0.0003
	Sucrose	0.3566 ± 0.0001
SWE 160	Xylose	0.3356 ± 0.0011
	Arabinose	0.3226 ± 0.0003
	Fructose	0.6339 ± 0.0002
	Glucose	0.9742 ± 0.0011
	Sucrose	0.2879 ± 0.0002
SWE 180	Arabinose	0.3373 ± 0.0004
	Fructose	0.4418 ± 0.0001
	Glucose	0.6988 ± 0.0002
SWE 200	Xylose	0.2822 ±0.0003
	Arabinose	0.3331 ± 0.0002
	Fructose	0.3589 ± 0.0003
	Glucose	0.6502 ± 0.0011
	Sucrose	0.2630 ± 0.0002
SWE 220	Xylose	0.2764 ± 0.0002
	Arabinose	0.2993 ± 0.0006
	Fructose	0.3116 ± 0.0003
	Glucose	0.4780 ± 0.0002
	Sucrose	0.2696 ± 0.0005
PEE 120	Fructose	0.4697 ± 0.0006
	Glucose	0.5234 ± 0.0004
	Sucrose	0.4191 ± 0.0004
PEE 140	Fructose	0.4465 ± 0.0003
	Glucose	0.5701 ± 0.0002
	Sucrose	0.4161 ± 0.0003
PEE 160	Xylose	0.3380 ± 0.0004
	Arabinose	0.3228 ± 0.0002
	Fructose	0.4393 ± 0.0002
	Glucose	0.4950 ± 0.0001
	Sucrose	0.3582 ± 0.0003
PEE 180	Fructose	0.5010 ± 0.0002
	Glucose	0.6298 ± 0.0002
PEE 200	Fructose	0.3903 ± 0.0002
	Glucose	0.5043 ± 0.0002
PEE 220	None detected	
UAE 20	Arabinose	0.2793 ± 0.0005
	Mannose	0.4242 ± 0.0002
	Galactose	0.4731 ± 0.0004
UAE 60	Xilose	0.0545 ± 0.0011
	Arabinose	0.1153 ± 0.0006
	Mannose	0.1632 ± 0.0001
	Galactose	0.4215 ± 0.0002
UAE 100	Xylose	0.3403 ± 0.0001
	Arabinose	0.0728 ± 0.0003
	Mannose	0.1055 ± 0.0002
	Galactose	0.4156 ± 0.0002
SFE100-UAE20	Fructose	0.1078 ± 0.0001
	Glucose	0.0824 ± 0.0003
	Sucrose	0.4751 ± 0.0003
	Maltose	0.0629 ± 0.0002
SFE100-UAE60	Fructose	0.2174 ± 0.0001
	Glucose	0.1652 ± 0.0002
	Sucrose	0.6929 ± 0.0003
	Maltose	0.0860 ± 0.0005
SFE100-UAE100	Fructose	0.1602 ± 0.0002
	Glucose	0.3325 ± 0.0003
	Sucrose	0.5050 ± 0.0001
	Maltose	0.0941 ± 0.0003
SFE200-UAE20	Xylose	0.0037 ± 0.0006
	Fructose	0.5889 ± 0.0002
	Glucose	0.2389 ± 0.0002
	Sucrose	0.4750 ± 0.0001
	Maltose	0.0716 ± 0.0004
SFE200-UAE60	Xylose	0.0534 ± 0.0017
	Fructose	0.1352 ± 0.0001
	Glucose	0.1024 ± 0.0001
	Sucrose	0.3753 ± 0.0003
	Maltose	0.0815 ± 0.0011
SFE200-UAE100	Fructose	0.1055 ± 0.0004
	Glucose	0.0641 ± 0.0011
	Sucrose	0.2832 ± 0.0003
	Maltose	0.0749 ± 0.0005
SFE300-UAE20	Fructose	0.2355 ± 0.0002
	Glucose	0.1300 ± 0.0011
	Sucrose	0.7181 ± 0.0004
	Maltose	0.1107 ± 0.0004
SFE300-UAE60	Rhamnose	0.0032 ± 0.0004
	Xylose	0.0032 ± 0.0006
	Arabinose	0.0152 ± 0.0004
	Fructose	0.1346 ± 0.0002
	Glucose	0.0933 ± 0.0005
	Sucrose	0.5207 ± 0.0001
	Maltose	0.0770 ± 0.0003
SFE300-UAE100	Fructose	0.0652 ± 0.0017
	Glucose	0.0452 ± 0.0011
	Sucrose	0.2031 ± 0.0002
	Maltose	0.0751 ± 0.0002
MAC	Fructose	0.4580 ± 0.0003
	Glucose	0.8681 ± 0.0001
	Sucrose	0.1722 ± 0.0003

All samples were analyzed in triplicate (*n* = 3). The results are expressed as mean values ± standard deviation (SD). Numbers indicate process parameters: UAE—amplitude (%); SFE—pressure (bar); SWE/PEE—temperature (°C). SWE—subcritical water extraction; PEE—pressurized ethanol extraction; UAE—ultrasound-assisted extraction; SFE-UAE—solid residues—ultrasound-assisted extraction; MAC—maceration.

**Table 5:** Antimicrobal activity of OPD extracts.

Microrganisam/ Sample	PEE 120 °C	PEE 140 °C	PEE 160 °C	UAE 20%	UAE 60%	UAE 100%
*Fusarium graminearum*	0	23.33	24.67	35	36.67	38
*Fusarium avenaceum*	0	18.33	18	20.67	24	24.33
*Alternaria alternata*	0	0	0	18.67	24.33	24
*Aspergillus flavus*	0	0	0	0	0	0
*Bacilluscereus*	7.33	0	0	12.67	11	11.33
*Salmonella enterica*	0	0	0	0	0	0

The antimicrobial activity results are presented as inhibition zone diameters in millimeters (mm). Results are expressed as mean values of three independent replicates (n = 3). PEE—pressurized ethanol extraction; UAE—ultrasound-assisted extraction.

**Table 6:** Comparative overview of selected green extraction techniques applied to agro-industrial by-products and orange peel dust (OPD).

Extraction Technique	Matrix	Extraction Conditions	Key Outcome
SWE	Chestnut exocarp [64]	120–220 °C, water	Highest antioxidant activity observed at 220 °C while 120 °C showed the strongest α-amylase/α-glucosidase inhibition
SWE	OPD	120–220 °C, water	Antioxidant activity increased with temperature (highest at 220 °C), and α-amylase inhibition peaked at 62.96% at 160 °C
PEE	Pisco grape pomace [65]	54% EtOH, 113 °C, 3 cycles	Pressurized hydroethanolic extraction resulted in high antioxidant capacity, outperforming conventional extraction.
PEE	OPD	120–220 °C, 50% EtOH	Antioxidant activity increased with temperature (highest at 220 °C), whereas α-amylase inhibition peaked at 37.36% at 160 °C and decreased at higher temperatures
UAE	*Aronia melanocarpa* pomace [66]	20–60% amplitude; 70% EtOH and water	UAE enabled efficient recovery of phenolic compounds with high antioxidant activity, with optimal performance observed at moderate ultrasound intensity; antimicrobial activity was also detected.
UAE	OPD	20–100% amplitude, 50% EtOH	Antioxidant activity showed a slight increase with ultrasound amplitude, while α-amylase inhibition remained high across all conditions (up to 59.05%); antimicrobial activity was observed, including antifungal and selective antibacterial effects.
SFE–UAE	Grapefruit, lime, and lemon peels [67]	Sc-CO_2_ followed by UAE with NaDES	Sequential extraction enabled broader valorization of citrus peel matrices, with complementary recovery of bioactive compounds depending on the applied extraction step
SFE–UAE	OPD	Sc-CO_2_ followed by UAE	Sequential SFE–UAE enhanced functional recovery, with the highest α-amylase inhibition observed among all techniques (up to 79.47%), while antioxidant activity remained moderate.

Values from the present study are expressed as mM TEAC/100 g dry sample (antioxidant, DPPH) and % inhibition (α-amylase); values from cited literature are expressed in different units depending on methodology (DPPH IC_50_, µmol TE/g DW, mg GAE/g). Direct numerical comparison is limited and should be interpreted qualitatively. Antimicrobial results are summarized qualitatively because the compared studies used different microorganisms and assay formats. SWE: subcritical water extraction; PEE: pressurized ethanol extraction; PLE: pressurized liquid extraction; UAE: ultrasound-assisted extraction; SFE: supercritical fluid extraction; Sc-CO_2_: supercritical carbon dioxide; NaDES: natural deep eutectic solvents.

## Data Availability

The original contributions presented in this study are included in the article. Further inquiries can be directed to the corresponding author.

## Abbreviations

The following abbreviations are used in this manuscript:

OPD	Orange peel dust
SWE	Subcritical water extraction
PEE	Pressurized ethanol extraction
UAE	Ultrasound-assisted extraction
Sc-CO_2_	Supercritical carbon dioxide extraction
MAC	Maceration
DPPH	2,2-diphenyl-1-picryhydrazyl
ABTS	2,2′-azino-bis-(3-ethylbenzothiazoline-6-sulfonic acid)
RP	Reducing power
